# Development of Diagnostics for Chagas Disease: Where Should We Put Our Limited Resources?

**DOI:** 10.1371/journal.pntd.0005148

**Published:** 2017-01-05

**Authors:** Albert Picado, Andrea Angheben, Andrea Marchiol, Belkisyolé Alarcón de Noya, Laurence Flevaud, Maria Jesus Pinazo, Montserrat Gállego, Sheba Meymandi, Silvia Moriana

**Affiliations:** 1 Foundation for Innovative New Diagnostics (FIND), Geneva, Switzerland; 2 Centre for Tropical Diseases, Hospital Sacro Cuore, Don Calabria, Negrar, Verona, Italy; 3 Drugs for Neglected Diseases initiative (DNDi), Geneva, Switzerland; 4 Immunology Section, Instituto de Medicina Tropical, Facultad de Medicina, Universidad Central de Venezuela, Caracas, Venezuela; 5 Médecins Sans Frontières Operational Centre Barcelona-Athens (MSF OCBA), Barcelona, Spain; 6 ISGlobal, Barcelona Ctr. Int. Health Res. (CRESIB), Hospital Clínic-Universitat de Barcelona, Barcelona, Spain; 7 Secció de Parasitologia, Departament de Biologia, Sanitat i Medi Ambient, Facultat de Farmàcia, Universitat de Barcelona, Barcelona, Spain; 8 Department of Cardiology, Center of Excellence for Chagas Disease at Olive View-UCLA Medical Center, Sylmar, California, United States of America; 9 The Global Chagas Disease Coalition, Barcelona, Spain; US Food and Drug Administration, UNITED STATES

According to the World Health Organization (WHO), one of the milestones set by the London Declaration for Chagas disease—the interruption of Chagas disease transmission through blood transfusion in Latin America—was achieved in most countries (20 out of 21) in 2015 [1]. This is a crucial step towards reaching the goal of controlling Chagas disease by 2020. However, many challenges remain: less than 1% of the 6 to 7 million people infected with *Trypanosoma cruzi* are treated, and new infections still occur [2].

Twenty to thirty percent of chronic Chagas disease patients will develop cardiac and/or gastrointestinal complications [3]. The WHO recommends treating *T*. *cruzi*-infected patients. Antiparasitic treatments are highly effective in patients in the acute phase and reduce the risk of disease progression in patients in the indeterminate stage of the disease (patients chronically infected with *T*. *cruzi* but without evidence of cardiac or gastrointestinal disease) [4,5]. Because *T*. *cruzi* can be transmitted by a variety of routes [6] (i.e., vectorial transmission when *T*. *cruzi* parasites, which are released when the infected blood-sucking triatomine bugs defecate, enter the body via the skin break caused by the bug’s bite or via other mucosa [e.g., oral transmission through contaminated food]; congenital infection; blood transfusion; and cell, blood, or tissue transplantation) and because the majority of people with chronic infection have never been tested and are not aware of their status, the development of new tools to diagnose Chagas disease is a priority. This is true in endemic countries as well as in regions where infected people have migrated in recent years (e.g., Europe, Asia, and North America). Despite concerted efforts to use available tools to diagnose Chagas disease in different patient groups and epidemiological and clinical settings in Latin American countries over the last four decades, and in nonendemic countries since the early 2000s, a large number of patients are diagnosed late or not at all. While the reasons for these diagnostic trends are diverse and vary across countries and patient groups, one of the main limitations is the lack of reliable diagnostic tests adapted to the needs of patients and health systems.

The resources to develop new diagnostic tools for Chagas disease are scarce, so it is important to allocate them to diagnostic needs that are not adequately covered by existing tools. Agreeing on diagnostic priorities will help to ensure that efforts and resources are directed to the development of tests that increase access to diagnosis and contribute to disease control. In recent years, the WHO, the Pan American Health Organization (PAHO), and Chagas disease experts have initiated this important process by identifying a range of diagnostic needs [7–11] (summarised in Table 1). The next step is for the Chagas disease community, including professionals with different backgrounds, expertise, and geographical locations, to rank these diagnostic needs.

**Table 1:** Diagnostic needs for Chagas disease.

Diagnostic need	Definition
Point-of-care* (PoC) test for Chagas disease patients in the acute phase	PoC test to identify acute *T*. *cruzi* infection (e.g., vector, oral transmission in the general population, reactivation in immunosuppressed patients).
PoC test for Chagas disease patients in the chronic phase	PoC test to identify chronic *T*. *cruzi* infection in the general population (including pregnant women).
Test to screen blood and organ donors/donations	Test to screen for *T*. *cruzi* infection in blood and organ donors/donations.
PoC test for congenital Chagas disease	PoC test to detect *T*. *cruzi* in newborns (as a result of congenital transmission of Chagas Disease).
Test for early assessment of treatment response	Test to assess efficacy of antiparasitic treatment soon after completion of treatment in the chronic phase.
Progression test: Test to identify patients with a high risk of developing Chagas disease complications	Test to identify, among *T*. *cruzi* infected patients, individuals with a high risk of developing neurologic, cardiac, or intestinal complications.
Test for early assessment of heart damage in Chagas disease patients	Test to identify, among *T*. *cruzi* infected patients, those with early cardiac damage.
Test for early assessment of digestive damage in Chagas disease patients	Test to identify, among *T*. *cruzi* infected patients, those with early digestive damage.
Test for screening of drug resistance to benznidazole and/or nifurtimox	Test to determine if *T*. *cruzi* is resistant to the available drugs (benznidazole/nifurtimox).

*A point-of-care (PoC) test is a test performed and interpreted where health care is provided, close to or near the patient.

To facilitate this ranking of diagnostic needs, we invited 155 Chagas disease experts to identify the three main diagnostic priorities for Chagas disease from Table 1. The experts were selected from among lead authors of Chagas disease scientific papers, physicians managing Chagas disease patients, and representatives of institutions involved in Chagas disease management (i.e., ministries of health, nongovernmental organizations [NGOs], private-public partnerships [PPP], the WHO/PAHO, and industry). Eighty-six of the experts (55%) were from Latin American countries; the other 69 (45%) were from nonendemic countries. Details on the 155 experts invited to participate are provided in S1 Table. The experts were asked to take into account the following: (1) existing diagnostic tools and (2) the expected clinical and epidemiological scenario of Chagas disease in the next five years. The survey was conducted in English, Spanish, and Portuguese in May and June of 2016 using Google Forms. For each expert, 3 points, 2 points, and 1 points were given to the first-, second-, and third-most-important priorities, respectively. A final score, calculated for each diagnostic need, was used to rank them. The results of the survey are presented here, pooled as well as by subgroup.

Sixty-two experts (40%) completed the survey; the respondents were equally distributed (*n* = 31) between Latin American and non-Latin American countries. The majority worked in research (*n* = 22) and hospitals or NGOs (*n* = 16), but respondents also included representatives of health ministries and the WHO/PAHO (*n* = 9) and patient associations (*n* = 1), among others (see S1 Table for details). The response rate was higher among experts from non-Latin American countries (45%) compared to Latin American experts (36%). As detailed in Table 2, four diagnostic needs obtained similar scores: (1) a test for early assessment of treatment response (score 83); (2) a point-of-care (PoC) test for congenital Chagas disease (score 76); (3) a progression test to identify patients with a high risk of developing Chagas disease complications (score 73); and (4) a PoC test for Chagas disease patients in the chronic phase (score 67). The different subgroups (e.g., Latin American versus non-Latin American experts, different areas of expertise) consistently identified the same four diagnostic priorities for Chagas disease.

**Table 2:** Ranking diagnostic needs for Chagas disease (June 2016).

Diagnostic need	Total	Latin America	Non-Latin America	Researchers	Hospitals/NGOs	MoH—WHO/PAHO^1^
Number of respondents	62	31	31	22	16	9
	Rank (score)	Rank (score)	Rank (score)	Rank (score)	Rank (score)	Rank (score)
Test for early assessment of treatment response	1 (83)	3 (35)	1 (48)	2 (25)	1 (26)	2 (14)
PoC^2^ test for congenital Chagas disease	2 (76)	1 (38)	3 (38)	3 (24)	4 (16)	1 (15)
Progression test: test to identify patients with a high risk of developing Chagas disease complications	3 (73)	4 (29)	2 (44)	1 (29)	2 (24)	4 (5)
PoC test for Chagas disease patients in the chronic phase	4 (67)	2 (36)	4 (31)	4 (18)	3 (19)	3 (12)

^1^MoH, Ministry of Health; WHO, World Health Organization; PAHO, Pan American Health Organization;

^2^PoC, Point of care

Experts from Latin America ranked the PoC test for congenital Chagas disease (score 38) as their top priority, whilst those from non-Latin American countries ranked the test for early assessment of treatment response (score 48) in first place. Researchers identified the progression test as the first priority, unlike respondents working in hospitals, NGOs, health ministries, and the WHO/PAHO, for whom the test to assess treatment response was the first priority. The rest of the diagnostic needs listed had lower scores (ranging from 1 to 28), and there were no major differences between subgroups, with one exception: the score for the PoC test for Chagas disease patients in the acute phase received a relatively high score from participants in Latin America (score 26) compared to non-Latin American experts (score 2). The complete dataset, including the scores for all diagnostic needs, is provided as supplementary material (S1 and S2 Tables).

According to respondents, the diagnostic tools currently available meet the requirements of some diagnostic needs, e.g., screening blood and organ donors or diagnosing Chagas disease patients in the acute phase. However, new diagnostic tools should be developed to assess treatment response, diagnose congenital Chagas disease, identify individuals at risk of developing Chagas disease-related complications, and diagnose *T*. *cruzi* infected individuals in the chronic phase. The development of those tools should be guided by detailed Target Product Profiles (TPPs) developed and endorsed by the WHO and the Chagas disease community. The current TPPs for Chagas disease diagnostics [8,12] should be reviewed and expanded to ensure they cover the priorities identified in this survey. The results of this survey and the revised TPPs should guide research groups and attract public and private funders interested in developing diagnostic tools for Chagas disease with the highest public health impact.

Defining the diagnostic needs and priorities for Chagas disease should be a dynamic process that is open to the whole Chagas disease community. To maximise input, the form used to collect the data presented in this paper will remain available at http://goo.gl/forms/66jt8cLxShAyXbm33 for six months from the date of publication.

## Supporting Information

S1 Table Individual ranking of the nine diagnostic needs for Chagas disease conducted by 62 experts. For each expert, 3 points, 2 points, and 1 point were given to the first, second, and third priorities, respectively.
(DOCX)

S2 Table Ranking and scores for all diagnostic needs for Chagas Disease. (DOCX)
