# Supplementary material for: Development of Diagnostics for Chagas Disease: Where Should We Put Our Limited Resources?
Source: PLoS Negl Trop Dis. 2017 Jan 5;11(1):e0005148. doi: 10.1371/journal.pntd.0005148 (PMC5221646; doi:10.1371/journal.pntd.0005148)
Supplement: S1 Table — For each expert, 3 points, 2 points, and 1 point were given to the first, second, and third priorities, respectively. (DOCX) [file pntd.0005148.s001.docx]

**S1 Table:** Individual ranking of the 9 diagnostic needs for Chagas disease conducted by 62 experts. For each expert, 3, 2 and 1 points were given to the first, second and third priorities respectively. The list with the details of non-respondents is added at the end of the table. PoC: Point of Care

| **type institution** | **Country** | **Latin America** | **PoC Acute** | **PoC Chronic** | **Screen Donors** | **PoC Congenital** | **Treat Response** | **Progression** | **Heart damage** | **Digestive Damage** | **drug resistance** |
| --- | --- | --- | --- | --- | --- | --- | --- | --- | --- | --- | --- |
| WHO/PAHO | Switzerland | no |  |  |  | 2 | 3 | 1 |  |  |  |
| Public-Private Partnership | Switzerland | no |  | 2 |  | 3 | 1 |  |  |  |  |
| Hospital | France | no |  | 3 |  |  | 1 | 2 |  |  |  |
| NGO | France | no |  | 1 |  |  | 2 | 3 |  |  |  |
| Research institute / University | Italy | no |  |  | 3 | 2 |  | 1 |  |  |  |
| Hospital | Italy | no | 1 |  |  | 3 |  | 2 |  |  |  |
| Hospital | Switzerland | no |  |  |  | 1 | 3 | 2 |  |  |  |
| Research institute / University | Brazil | yes |  |  |  | 1 |  | 2 | 3 |  |  |
| Research institute / University | USA | no |  | 3 |  | 2 |  | 1 |  |  |  |
| Research institute / University | Brazil | yes | 2 | 1 |  |  | 3 |  |  |  |  |
| Research institute / University | USA | no |  | 3 |  |  | 1 | 2 |  |  |  |
| Research institute / University | Bolivia | yes | 3 | 2 |  | 1 |  |  |  |  |  |
| Research institute / University | Italy | no |  | 3 |  | 2 | 1 |  |  |  |  |
| Research institute / University | Brazil | yes |  |  |  |  | 3 | 1 |  |  | 2 |
| Hospital | Italy | no |  | 2 |  |  |  | 3 | 1 |  |  |
| Research institute / University | Brazil | yes | 3 | 2 | 1 |  |  |  |  |  |  |
| Research institute / University | USA | no |  |  |  | 3 |  |  | 1 |  | 2 |
| NGO | Italy | no |  | 3 |  |  | 2 | 1 |  |  |  |
| Research institute / University | USA | no |  |  |  |  | 3 | 2 |  |  | 1 |
| Diagnostic laboratory | Netherlands | no | 1 |  |  |  | 3 | 2 |  |  |  |
| Ministry of Health | Chile | yes |  |  |  |  | 3 | 2 |  |  | 1 |
| Hospital | Spain | no |  | 1 |  | 2 | 3 |  |  |  |  |
| Diagnostic laboratory | Honduras | yes |  | 2 |  | 3 |  |  | 1 |  |  |
| Patients Association | Mexico | yes | 2 | 3 |  | 1 |  |  |  |  |  |
| Hospital | Spain | no |  |  |  | 3 | 2 |  |  |  | 1 |
| Hospital | Spain | no |  | 3 |  |  | 2 |  | 1 |  |  |
| Public-Private Partnership | Mexico | yes |  | 2 | 1 | 3 |  |  |  |  |  |
| Research institute / University | Venezuela | yes |  |  |  |  | 2 | 3 |  |  | 1 |
| Hospital | Spain | no |  |  |  |  | 2 | 3 |  |  | 1 |
| Hospital | Argentina | yes |  | 1 |  |  | 3 | 2 |  |  |  |
| Diagnostic laboratory | Spain | no |  |  |  | 3 | 2 | 1 |  |  |  |
| Diagnostic laboratory | Spain | no |  | 3 |  |  |  |  | 2 | 1 |  |
| Research institute / University | Spain | no |  |  |  | 2 |  | 3 |  |  | 1 |
| Research institute / University | Paraguay | yes |  |  |  | 2 | 1 | 3 |  |  |  |
| NGO | Ecuador | yes |  | 3 |  | 2 | 1 |  |  |  |  |
| Ministry of Health | Argentina | yes |  |  |  | 3 | 2 | 1 |  |  |  |
| Research institute / University | Ecuador | yes |  |  |  | 3 | 2 |  | 1 |  |  |
| Research institute / University | Colombia | yes | 3 |  |  | 2 |  | 1 |  |  |  |
| Hospital | Spain | no |  |  |  |  |  | 3 | 2 |  | 1 |
| Research institute / University | Uruguay | yes |  |  | 3 | 2 | 1 |  |  |  |  |
| Others | Brazil | yes | 2 | 3 |  | 1 |  |  |  |  |  |
| Research institute / University | Bolivia | yes |  |  |  |  | 2 | 3 |  |  | 1 |
| Public-Private Partnership | Bolivia | yes | 2 |  |  | 3 | 1 |  |  |  |  |
| Public-Private Partnership | Argentina | yes |  | 1 |  | 3 | 2 |  |  |  |  |
| Hospital | Honduras | yes | 3 | 2 |  | 1 |  |  |  |  |  |
| Ministry of Health | Mexico | yes |  | 3 |  |  | 2 | 1 |  |  |  |
| Research institute / University | Brazil | yes |  | 3 |  |  |  | 2 | 1 |  |  |
| Ministry of Health | Brazil | yes | 3 |  |  |  | 2 |  | 1 |  |  |
| Research institute / University | Brazil | yes |  | 1 |  |  | 3 | 2 |  |  |  |
| Research institute / University | Brazil | yes |  |  |  |  |  | 3 | 2 |  | 1 |
| Diagnostic laboratory | Spain | no |  |  |  | 1 | 3 |  |  |  | 2 |
| Industry | France | no |  |  |  |  | 2 | 3 |  |  | 1 |
| Industry | France | no |  |  | 1 |  | 2 | 3 |  |  |  |
| Industry | France | no |  | 2 |  |  | 1 | 3 |  |  |  |
| Hospital | Switzerland | no |  |  |  | 3 | 2 | 1 |  |  |  |
| Research institute / University | Belgium | no |  |  |  | 2 | 3 |  |  |  | 1 |
| Public-Private Partnership | Brazil | yes |  |  |  |  | 1 | 3 |  |  | 2 |
| Ministry of Health | Brazil | yes |  | 2 |  | 3 | 1 |  |  |  |  |
| Ministry of Health | Colombia | yes | 2 | 3 |  | 1 |  |  |  |  |  |
| WHO/PAHO | USA | no |  | 2 |  | 3 | 1 |  |  |  |  |
| Hospital | Spain | no |  |  |  | 1 | 3 | 2 |  |  |  |
| Ministry of Health | Colombia | yes | 1 | 2 |  | 3 |  |  |  |  |  |
| Hospital | Brazil | yes | Non respondent |  |  |  |  |  |  |  |  |
| Research institute / University | Argentina | yes | Non respondent |  |  |  |  |  |  |  |  |
| Research institute / University | Italy | no | Non respondent |  |  |  |  |  |  |  |  |
| Ministry of Health | Chile | yes | Non respondent |  |  |  |  |  |  |  |  |
| Research institute / University | Brazil | yes | Non respondent |  |  |  |  |  |  |  |  |
| Patients association | Brazil | yes | Non respondent |  |  |  |  |  |  |  |  |
| WHO/PAHO | USA | no | Non respondent |  |  |  |  |  |  |  |  |
| Research institute / University | USA | no | Non respondent |  |  |  |  |  |  |  |  |
| Research institute / University | Venezuela | yes | Non respondent |  |  |  |  |  |  |  |  |
| Industry | Korea | no | Non respondent |  |  |  |  |  |  |  |  |
| Public-Private Partnership | Brazil | yes | Non respondent |  |  |  |  |  |  |  |  |
| Ministry of Health | Paraguay | yes | Non respondent |  |  |  |  |  |  |  |  |
| Ministry of Health | Honduras | yes | Non respondent |  |  |  |  |  |  |  |  |
| Research institute / University | Bolivia | yes | Non respondent |  |  |  |  |  |  |  |  |
| Research institute / University | Brazil | yes | Non respondent |  |  |  |  |  |  |  |  |
| NGO | Argentina | yes | Non respondent |  |  |  |  |  |  |  |  |
| Ministry of Health | Argentina | yes | Non respondent |  |  |  |  |  |  |  |  |
| Ministry of Health | Argentina | yes | Non respondent |  |  |  |  |  |  |  |  |
| Ministry of Health | Bolivia | yes | Non respondent |  |  |  |  |  |  |  |  |
| Ministry of Health | Argentina | yes | Non respondent |  |  |  |  |  |  |  |  |
| Ministry of Health | Guatemala | yes | Non respondent |  |  |  |  |  |  |  |  |
| Research institute / University | Brazil | yes | Non respondent |  |  |  |  |  |  |  |  |
| Research institute / University | Venezuela | yes | Non respondent |  |  |  |  |  |  |  |  |
| Research institute / University | Mexico | yes | Non respondent |  |  |  |  |  |  |  |  |
| NGO | Italy | no | Non respondent |  |  |  |  |  |  |  |  |
| Ministry of Health | Mexico | yes | Non respondent |  |  |  |  |  |  |  |  |
| NGO | Argentina | yes | Non respondent |  |  |  |  |  |  |  |  |
| Public-Private Partnership | Brazil | yes | Non respondent |  |  |  |  |  |  |  |  |
| WHO/PAHO | USA | no | Non respondent |  |  |  |  |  |  |  |  |
| Research institute / University | Colombia | yes | Non respondent |  |  |  |  |  |  |  |  |
| Research institute / University | Argentina | yes | Non respondent |  |  |  |  |  |  |  |  |
| Research institute / University | Italy | no | Non respondent |  |  |  |  |  |  |  |  |
| Research institute / University | Paraguay | yes | Non respondent |  |  |  |  |  |  |  |  |
| Research institute / University | Ecuador | yes | Non respondent |  |  |  |  |  |  |  |  |
| hospital | Argentina | yes | Non respondent |  |  |  |  |  |  |  |  |
| Ministry of Health | Argentina | yes | Non respondent |  |  |  |  |  |  |  |  |
| Hospital | Argentina | yes | Non respondent |  |  |  |  |  |  |  |  |
| Research institute / University | Mexico | yes | Non respondent |  |  |  |  |  |  |  |  |
| Industry | Spain | no | Non respondent |  |  |  |  |  |  |  |  |
| Research institute / University | Colombia | yes | Non respondent |  |  |  |  |  |  |  |  |
| Research institute / University | Portugal | no | Non respondent |  |  |  |  |  |  |  |  |
| Patients association | Spain | no | Non respondent |  |  |  |  |  |  |  |  |
| Ministry of Health | El Salvador | yes | Non respondent |  |  |  |  |  |  |  |  |
| Ministry of Health | Panamá | yes | Non respondent |  |  |  |  |  |  |  |  |
| Ministry of Health | Bolivia | yes | Non respondent |  |  |  |  |  |  |  |  |
| Ministry of Health | Honduras | yes | Non respondent |  |  |  |  |  |  |  |  |
| Research institute / University | France | no | Non respondent |  |  |  |  |  |  |  |  |
| Ministry of Health | Venezuela | yes | Non respondent |  |  |  |  |  |  |  |  |
| NGO | Brazil | yes | Non respondent |  |  |  |  |  |  |  |  |
| Research institute / University | Italy | no | Non respondent |  |  |  |  |  |  |  |  |
| Research institute / University | Spain | no | Non respondent |  |  |  |  |  |  |  |  |
| Research institute / University | Spain | no | Non respondent |  |  |  |  |  |  |  |  |
| NGO | Argentina | yes | Non respondent |  |  |  |  |  |  |  |  |
| NGO | USA | no | Non respondent |  |  |  |  |  |  |  |  |
| Hospital | Bolivia | yes | Non respondent |  |  |  |  |  |  |  |  |
| Research institute / University | Spain | no | Non respondent |  |  |  |  |  |  |  |  |
| Research institute / University | Brazil | yes | Non respondent |  |  |  |  |  |  |  |  |
| Patients association | Spain | no | Non respondent |  |  |  |  |  |  |  |  |
| NGO | USA | no | Non respondent |  |  |  |  |  |  |  |  |
| Research institute / University | Uruguay | yes | Non respondent |  |  |  |  |  |  |  |  |
| Research institute / University | Venezuela | yes | Non respondent |  |  |  |  |  |  |  |  |
| Industry | Spain | no | Non respondent |  |  |  |  |  |  |  |  |
| NGO | Spain | no | Non respondent |  |  |  |  |  |  |  |  |
| Ministry of Health | Colombia | yes | Non respondent |  |  |  |  |  |  |  |  |
| Ministry of Health | Colombia | yes | Non respondent |  |  |  |  |  |  |  |  |
| Ministry of Health | Peru | yes | Non respondent |  |  |  |  |  |  |  |  |
| Research institute / University | UK | no | Non respondent |  |  |  |  |  |  |  |  |
| Industry | Italy | no | Non respondent |  |  |  |  |  |  |  |  |
| NGO | Spain | no | Non respondent |  |  |  |  |  |  |  |  |
| Research institute / University | Venezuela | yes | Non respondent |  |  |  |  |  |  |  |  |
| Ministry of Health | Costa Rica | yes | Non respondent |  |  |  |  |  |  |  |  |
| Ministry of Health | Nicaragua | yes | Non respondent |  |  |  |  |  |  |  |  |
| Research institute / University | Venezuela | yes | Non respondent |  |  |  |  |  |  |  |  |
| Research institute / University | USA | no | Non respondent |  |  |  |  |  |  |  |  |
| Research institute / University | Spain | no | Non respondent |  |  |  |  |  |  |  |  |
| Research institute / University | USA | no | Non respondent |  |  |  |  |  |  |  |  |
| Research institute / University | UK | no | Non respondent |  |  |  |  |  |  |  |  |
| Industry | France | no | Non respondent |  |  |  |  |  |  |  |  |
| Industry | USA | no | Non respondent |  |  |  |  |  |  |  |  |
| Research institute / University | Argentina | yes | Non respondent |  |  |  |  |  |  |  |  |
| Research institute / University | USA | no | Non respondent |  |  |  |  |  |  |  |  |
| Research institute / University | USA | no | Non respondent |  |  |  |  |  |  |  |  |
| WHO/PAHO | USA | no | Non respondent |  |  |  |  |  |  |  |  |
| Research institute / University | Brazil | yes | Non respondent |  |  |  |  |  |  |  |  |
| Research institute / University | Mexico | yes | Non respondent |  |  |  |  |  |  |  |  |
| WHO/PAHO | USA | no | Non respondent |  |  |  |  |  |  |  |  |
| Ministry of Health | Argentina | yes | Non respondent |  |  |  |  |  |  |  |  |
| Industry | Japan | no | Non respondent |  |  |  |  |  |  |  |  |
| Research institute / University | USA | no | Non respondent |  |  |  |  |  |  |  |  |
| Industry | USA | no | Non respondent |  |  |  |  |  |  |  |  |
| Research institute / University | Sweden | no | Non respondent |  |  |  |  |  |  |  |  |
| Research institute / University | France | no | Non respondent |  |  |  |  |  |  |  |  |
| Ministry of Health | Venezuela | yes | Non respondent |  |  |  |  |  |  |  |  |
