# Supplementary material for: Development of Diagnostics for Chagas Disease: Where Should We Put Our Limited Resources?
Source: PLoS Negl Trop Dis. 2017 Jan 5;11(1):e0005148. doi: 10.1371/journal.pntd.0005148 (PMC5221646; doi:10.1371/journal.pntd.0005148)
Supplement: S2 Table — (DOCX) [file pntd.0005148.s002.docx]

**S2 Table:** Ranking and Scores all diagnostic needs for Chagas disease.

|  | **N** | **PoC Acute** | **PoC Chronic** | **Screen Donors** | **PoC Congenital** | **Treat Response** | **Progression** | **Heart damage** | **Digestive Damage** | **drug resistance** |
| --- | --- | --- | --- | --- | --- | --- | --- | --- | --- | --- |
| **Score Total** | 62 | 28 | 67 | 9 | 76 | 83 | 73 | 16 | 1 | 19 |
| ranking Total |  |  | 4 |  | 2 | 1 | 3 |  |  |  |
|  |  |  |  |  |  |  |  |  |  |  |
| Score Latin America | 31 | 26 | 36 | 5 | 38 | 35 | 29 | 9 | 0 | 8 |
| ranking Latin America |  |  | 2 |  | 1 | 3 | 4 |  |  |  |
|  |  |  |  |  |  |  |  |  |  |  |
| Score Non-Latin America | 31 | 2 | 31 | 4 | 38 | 48 | 44 | 7 | 1 | 11 |
| ranking Non-Latin America |  |  | 4 |  | 3 | 1 | 2 |  |  |  |
|  |  |  |  |  |  |  |  |  |  |  |
| Score Researchers | 22 | 11 | 18 | 7 | 24 | 25 | 29 | 8 | 0 | 10 |
| ranking Researchers |  |  | 4 |  | 3 | 2 | 1 |  |  |  |
|  |  |  |  |  |  |  |  |  |  |  |
| Score Hospital/NGO | 16 | 4 | 19 | 0 | 16 | 26 | 24 | 4 | 0 | 3 |
| ranking Hospital/NGO |  |  | 3 |  | 4 | 1 | 2 |  |  |  |
|  |  |  |  |  |  |  |  |  |  |  |
| Score MoH/WHO/PAHO | 9 | 6 | 12 | 0 | 15 | 14 | 5 | 1 | 0 | 1 |
| ranking MoH/WHO/PAHO |  |  | 3 |  | 1 | 2 | 4 |  |  |  |

PoC: Point of Care
